# Reliability and Validity of the Chinese General Social Capital Scale and Its Effect on Physical Disease and Psychological Distress among Chinese Medical Professionals

**DOI:** 10.3390/ijerph18126635

**Published:** 2021-06-20

**Authors:** Sibo Zhao, Yanwen Li, Yonggang Su, Long Sun

**Affiliations:** 1 School of Sociology and Psychology, Central University of Finance and Economics, Beijing 100081, China; sibozhao@cufe.edu.cn; 2 Shandong Academy of Social Sciences, Jinan 250002, China; Leeyanwen163@163.com; 3 School of Basic Medical Sciences, Shandong University, Jinan 250012, China; syg@sdu.edu.cn; 4 Center for Health Management and Policy Research, School of Public Health, Cheeloo College of Medicine, Shandong University, 44 Wenhuaxi Road, Jinan 250012, China; 5 Key Laboratory for Health Economics and Policy Research, National Health Commission of China (Shandong University), Jinan 250012, China

**Keywords:** reliability, validity, general social capital scale, physical health, mental health

## Abstract

The study was designed with two objectives. The first was to assess the factor structure, internal consistency reliability, and preliminary psychometric properties of the Chinese version of the Chinese-translated General Social Capital Scale (GSCS) in a sample of Chinese medical professionals. The second was to investigate the association between general social capital, physical disease, and psychological distress using the same Chinese sample. The English version of the GSCS was translated into Chinese, and its factor structure, estimates of internal consistency reliability, and psychometric properties were examined in a representative sample of medical professionals. In particular, a total of 3367 participants in Shandong Province, China were identified using the multi-stage stratified sampling method. In addition to the GSCS, preliminary data were collected using self-report instruments that included questionnaires on physical diseases, psychological distress, and general sociodemographic information. Results include internal consistency reliability estimates at 0.933 and acceptable values of the Guttman split-half coefficients for the GSCS and its subscales. The Kaiser–Meyer–Olkin value for the Chinese GSCS was 0.933, and the *p*-value of Bartlett’s test was less than 0.001. Exploratory factor analysis supported nine components of the scale with an acceptable cumulative rate (66.63%). The study further found a negative relationship between physical diseases, psychological distress, and social capital. The Chinese version of the GSCS has a satisfactory factor structure, reliability estimates, and satisfactory evidence of concurrent validity estimates for medical professionals from various demographic backgrounds. The current scale holds promise for wide use in future investigations on Chinese populations.

## 1. Background

The association between social capital and health has gained increased research attention during the previous two decades. A large body of epidemiological literature has documented the association between individual social context and physical or mental health, with a special focus on the benefits of resources inherent in the structures of social capital for the prevention of physical disease and for the achievement of psychological well-being [1,2]. Studies on the mechanisms of the influence of social capital on health have generally found that low social capital (i.e., trust and social participation) increases the risk of hypertension, other illnesses, or self-reported health through lifestyle behaviors [3,4,5]. A large body of evidence using data across populations has found that social capital may be a protective factor in the prevention of mental illness [6,7,8,9]. In general, these studies concluded that social capital may lower the risk of psychological distress and increase resilience and subjective well-being at the same time [10,11]. Using a nationally representative sample of middle aged adults in the U.S., Fujiwara and Kawachi [12] examined various dimensions of social capital and their associations with risk of major depression after controlling for individual potential confounders. The results indicated that high levels of cognitive social capital (measured by trust) were associated with low risks of developing major depression because high levels of social trust within a community helps in the formation of health-related social norms, which may have a protective effect for major depression.

Although a considerable body of literature has addressed social capital and its associations with the risk of psychological distress and physical disease in Western countries, much less is known regarding the above relationships in China—a nation that has experienced rapid economic and industrial development over the past two decades. Compared to other countries, China’s economic development and social changes have brought about drastic changes in the social structure, involving changes in the social role and social status of individuals, particularly in the individual’s social capital.

Social capital as a social construct emerged by the late 20th century based on the works of Bourdieu [13] and Coleman [14] with emphasis on the provision of access to important assets for communities and individuals via social relationships. Although a universally accepted definition of social capital is missing, it embraces high consistency in terms of forms and dimensions at the general level. Researchers remain interested in several components of social capital such as available resources (Bourdieu) [13], social structures used to achieve desired goals [15], and social networks that unite individuals and enhance the development of norms, reciprocity, and trust [16].

Although social capital is developing as a theory rich in understanding the relationships between personal networking, societal norms, and social outcomes, debate continues over its characterization across disciplines. From the economic perspective, social capital is an individual’s accrual of social characteristics (i.e., social capital increases in occupations with greater returns to social skills and declines with expected mobility) [17]. However, sociology views social capital as a feature of a group of people such as social classes [18]. Based on a cross-national empirical analysis, Pichler and Wallace [19] revealed that the pattern of social capital reflected the stratification patterns of a society with the upper layers displaying high levels of social capital through social networks. In the same manner, countries with high levels of inequality magnified such differences between classes, thus giving the upper classes further advantages. Sociology also views social capital as embedded in groups and individuals, thus playing an important role in social norms and trust [14,20]. In this study, we adopted the latter point of view for further demonstration.

In general, the previous literature on social capital has provided large bodies of evidence and demonstrated that the greater the social capital, the better the health outcomes [21], and this relationship has attracted the attention of scholars across disciplines to study the mechanisms of health outcomes in a social context. One of the most discussed theoretical models within the field of occupational health is the Person-Environment Fit Model (P-E fit) [22], which highlights similarity or convergence between the attributes of a person and those of their environment. However, unlike what was found in Western countries, the P-E fit model might not be entirely applicable within Chinese society where people care more about how appropriately they act, rather than their congruence with the environment [23]. Hence, the P-E fit model is limited because it ignores the motivation and ability of human agency to manage fit based upon their existing resources [24]. Individuals with high social capital are expected to be associated with better life satisfaction and organizational citizenship behavior [25]. When social capital declines, it is often accompanied by negative social outcomes [26]. For instance, low social capital was associated with decreased job satisfaction in health professionals [27] and increased job tension during crisis [28].

Although the concept of social capital has been the focus of much research in theoretical and applied social sciences, a clarification of its measures remains unresolved [29] in terms of dimensions and contexts or fields [30]. Research has pointed out that no universally applicable tool is used to measure social capital, thus resulting in the inconclusive findings on social capital and its outcomes due to the use of competing scales [31]. The literature on the quantitative research on social capital has recognized the need to focus on a standard measure. However, concern has arisen over people with different occupational groups across countries. One self-report instrument that received increased attention from the extant literature on the assessment of community-based social capital is the general social capital scale (GSCS). Onyx and Bullen [32] developed a valid and practical scale to measure social capital based on five communities in Australia and investigated the reliability and validity of the scale. The GSCS is measured on a 4-point Likert-type scale ranging from 1 (no, not much or no, not at all) to 4 (yes, definitely or yes, frequently). The original scale comprised 36 items with eight specific independent factors, namely, participation in the local community, proactivity in the social context, feelings of trust and safety, neighborhood connections, family and friend connections, tolerance of diversity, value of life, and work connections. Onyx and Bullen (2000) demonstrated that the overall reliability of the scale reached a Cronbach’s alpha of 0.84, and total inter-correlations ranged from 0.25 to 0.45, thus confirming the psychometric strength of the original scale. The GSCS has been used worldwide in research on social capital across domains such as community governance [33] and the relationship between poverty and health [34]. However, given the circumstances of many measures of social capital that select specific items from existing social capital scales in a non-random manner [35], an effective and comparable tool for measuring social capital in occupational groups in China remains lacking. Thus, this should be urgently addressed.

In the context of China’s socio-economic transformation, there has been a considerable increase in public hospital reform, which has led to an escalation in demands on health care professionals. The combination of high demands and high risks, fierce market competition, high workload, and hospital–patient conflicts has created a strenuous and arduous environment for medical professionals and brought high pressure and massive burden to their social lives. A significant characteristic of the study is that it recognizes the need to develop the Chinese version of the GSCS by estimating reliability and validity as well as evaluating the construct of social capital among medical professionals in China. It also highlights the importance of investigating the link between social capital, physical diseases, and psychological distress in public health. Therefore, the current study presents its specific objectives as follows. First, the replicability of the GSCS in a large sample of medical professionals was examined. Second, the structure of the final solution was confirmed in the second half of the sample (referred to as cross-validation for analyses). Third, evidence of internal consistency reliability for scores on each domain was evaluated. Fourth, the correlates of the GSCS, physical diseases, and psychological distress were assessed. In this manner, the study hopes to further advance this field for scholars and practitioners by providing a set of empirical tools for measuring social capital and understanding its effect on physical diseases and psychological distress among occupational groups in the Chinese setting.

## 2. Methods

### 2.1. Sampling and Participants

The study recruited medical professionals (doctor, nurse, and medical technician) based on a cross-sectional design in Shandong Province, China. In Shandong Province, there were 341,971 medical professionals at the end of 2018 [36]. Multi-stage stratified cluster sampling was used to select the participants. First, all of the 17 cities in Shandong Province were classified into three groups according to GDP per capita for 2017. In each group, one city was randomly selected for interview, and three cities (Qingdao, Dezhou, and Zaozhuang) were instructed to complete the questionnaire. Second, three counties (districts) out of all counties (districts) from each city were randomly selected. Third, one city-level general hospital was randomly selected from each city and one county-level hospital from each county (districts). In total, 12 general hospitals from the three groups (three city-level hospitals and nine county-level hospitals) were selected. Fourth, three in-patient areas from each department were randomly selected for each city-level hospital. Moreover, two in-patient areas from each department in each county-level hospital were selected. All medical professionals working on the date of the interview were scheduled for the interview. Finally, a total of 3367 valid questionnaires with a response rate of 84.7% (3367/3852) were collected.

### 2.2. Data Collection

The survey was carried out from November 2018 to January 2019. After selecting the departments, questionnaires were individually sent to the medical professionals, and managers in the hospitals helped to dispense the questionnaires. The medical professionals were asked to fill out the questionnaires anonymously on days without work. Two trained post-graduate students facilitated the distribution in the hospitals, answered the questions, and collected the questionnaires as well as conducted inspection tours in each department to make sure that the participants filled out the questionnaire by themselves. The participants were not compensated for their participation in this study.

### 2.3. Generation of the Chinese Version of the GSCS

The original GSCS scale was published by Onyx and Bullen [32]. After receiving permission from the authors, the standard forward and backward procedure was followed for scale translation. First, one professor who majored in sociology was employed to translate the original English scale into Chinese (forward step). Second, another professor who majored in English was recruited to back-translate the draft to English (backward step). Third, the researchers compared the two English versions of the GSCS and modified the Chinese version to ensure the integrity and fluency of the questions. Finally, each researcher was required to read the final Chinese and English versions to ensure all information in the original scale was included in the Chinese version.

### 2.4. Measures

#### 2.4.1. Physical Health

Physical health was assessed via an item indicating if the medical professionals had been diagnosed with any chronic disease with a binary response: 1 = yes and 0 = no.

#### 2.4.2. Psychological Distress

The Kessler 10 (K10) scale was used to measure the psychological distress level of the medical professionals [37]. The scale features 10 items with a 5-point Likert-type rating scheme. It is widely used to evaluate psychological distress [38,39,40]. Previous studies have established the reliability and validity of the Chinese version of K10 [41]. The current study reached a Cronbach’s alpha of 0.932 for K10.

#### 2.4.3. Social-Demographic Variables

The social-demographic variables were classified according to gender, age (assessed by date of birth up to the date of the survey/interview), and marital status (single, married, divorced, widowed, and others). As few subjects were divorced or widowed, they were categorized into single, married, and others. Education was denoted as the highest academic degree received (PhD, master’s, bachelor’s, and others). Professional titles were categorized as senior, vice-senior, intermediate, and junior, and others. Managerial position was also determined.

### 2.5. Statistical Analysis

IBM SPSS Statistics 24.0 (Web Edition) (IBM, Armonk, NY, USA) and IBM SPSS AMOS (version 22.0) (IBM, Armonk, NY, USA) were used for data analysis. Internal consistency reliability and split-half reliability of the scale were calculated using SPSS Statistics and used for exploratory factor analysis (EFA). Confirmatory factor analysis (CFA) in SPSS AMOS was conducted to build the factor model. Logistic regression and linear regression in SPSS Statistics were conducted to explore the effect of social capital on physical diseases and mental health, respectively. All tests were two-tailed and a *p*-value < 0.05 was considered statistically significant.

## 3. Results

### 3.1. Sample Characteristics

To evaluate the reliability and validity of the Chinese version of the GSCS and analyze the association between physical diseases, mental health, and social capital, a total of 3367 medical professionals answered the questionnaire in Chinese general hospitals. The medical professionals consisted of doctors (*n* = 1244; 36.9%), nurses (*n* = 1699; 49.6%), and medical technicians (*n* = 454; 13.5%). Nurses were the dominant population in the study; thus the proportion of females (73.2%) was also higher than that of males (26.8%). Data on age, marital status, education, professional title, and managerial position were collected. In this sample, 449 (13.3%) medical professionals reported chronic disease. The mean of psychological distress was 22.17, with a SD 7.44. Table 1 provides a detailed description.

### 3.2. Reliability Evaluation of GSCS

In the current study, the internal consistency and split-half reliability for the Chinese version of the GSCS were calculated. As shown in Table 2, all Cronbach’s alpha values for the scale and subscales were higher than 0.65, which indicated positive internal consistency reliability. In terms of split-half reliability, the Guttman split-half coefficient values for the scale and subscales were higher than 0.60, which indicated that the Chinese version of the GSCS had acceptable split-half reliability.

### 3.3. Validity Evaluation of GSCS

Prior to EFA, the Kaiser–Meyer–Olkin (KMO) value and Bartlett’s test were calculated. Results showed that the KMO value for the Chinese GSCS was 0.933, and the *p*-value of Bartlett’s test was less than 0.001. Thus, both results indicated that EFA could be used in the database.

Table 3 displays the EFA results for the Chinese version of the GSCS with nine components reaching an acceptable cumulative rate (66.63%). Compared with the original English scale, the first difference was the factor of *feelings of trust and safety*. With an explanation of the items in this factor, we divided the two factors into *feeling of trust* and *feeling of safety*. The second difference was noted for 13, whose factor loadings were similar to factors 2 and 9. In the same manner, item 13 was more similar to the items in factor 2.

**Figure 1:**
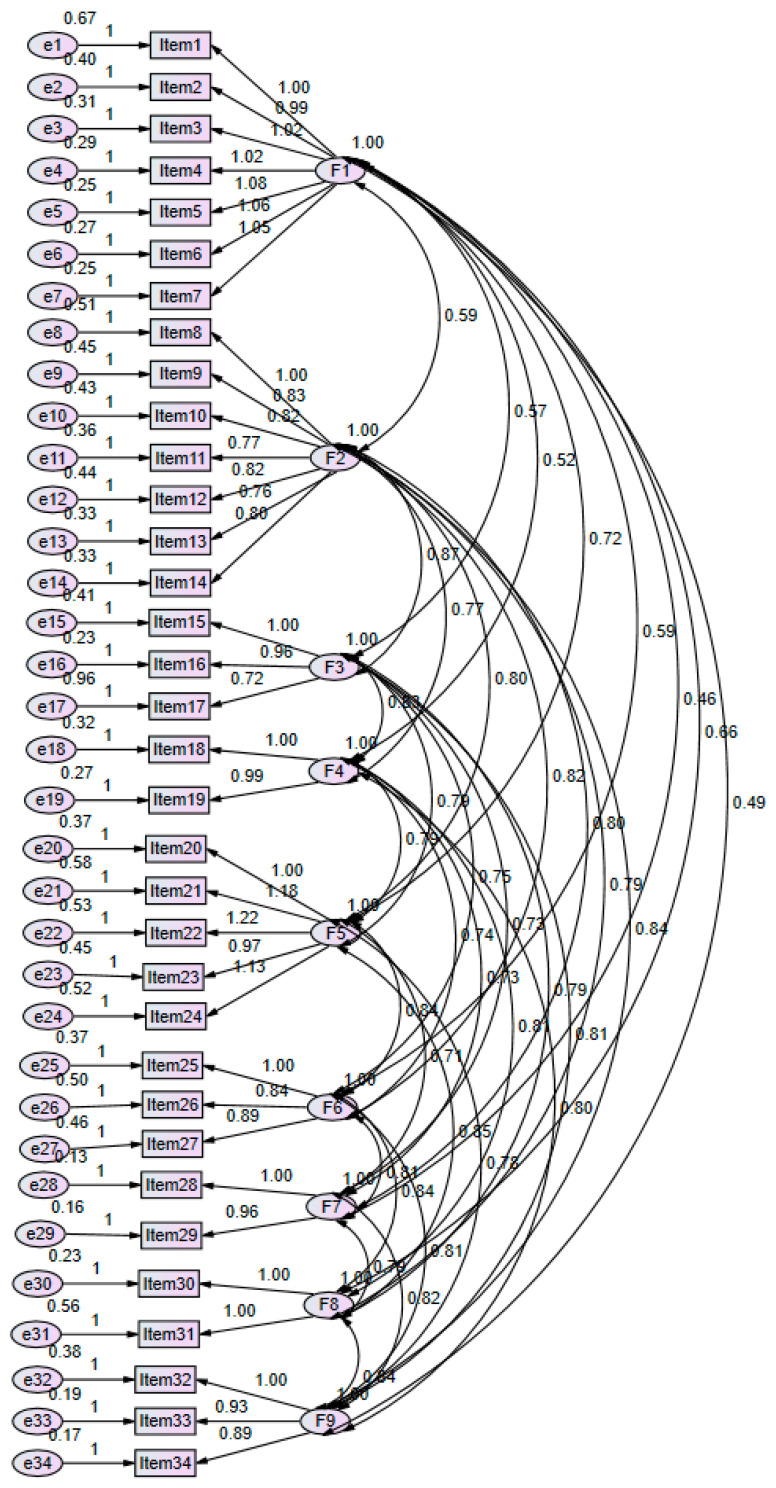
The detailed coefficients for the factor structure of the GSCS-Chinese version.

To ensure the structure validity of the Chinese version of the GSCS, CFA was employed to analyze the database. The study built a nine-factor structure in the model. Results implied that the model fit was acceptable for the Chinese version of the GSCS (RMSEA = 0.071, GFI = 0.851, AGFI = 0.823). Figure 1 illustrates the detailed coefficients.

### 3.4. Effect of General Social Capital on Physical Diseases

After evaluating the reliability and validity of the Chinese version of the GSCS, the study further analyzed the association between physical diseases and social capital. Results indicated that high levels of social capital were more likely associated with physical diseases (OR = 0.98, *p* < 0.001). Other risk factors were old age (OR = 1.05; *p* < 0.001), bachelor’s degree (OR = 1.67, *p* = 0.010), senior professional title (OR = 2.41; *p* = 0.006), vice-senior professional title (OR = 1.99; *p* = 0.003), and intermediate professional title (OR = 1.67, *p* = 0.001). Table 4 provides the detailed information.

### 3.5. Effect of General Social Capital on Psychological Distress

Moreover, the association between social capital and psychological distress was analyzed. Results indicated that high levels of social capital were negatively associated with psychological distress (β = −0.28, *p* < 0.001). Other related factors were male (β = 0.04, *p* = 0.027), old age (β = 0.06, *p* = 0.022), single marital status (β = −0.05, *p* = 0.022), PhD (β = 0.05, *p* = 0.035), master’s degree (β = 0.05, *p* = 0.029), bachelor’s degree (β = 0.11, *p* < 0.001), and senior professional title (β = 0.10, *p* < 0.001). Table 5 presents the detailed information.

## 4. Discussion

The current study mainly aimed to evaluate the reliability and validity of the Chinese version of the GSCS and analyzed the associations between physical diseases, psychological distress, and social capital among medical professionals. Results support the notion that the GSCS has acceptable reliability and validity among Chinese medical professionals, where high levels of social capital are negatively associated with physical diseases and psychological distress.

The first aim of the current study was to evaluate the reliability and validity of the Chinese version of GSCS. In recent years, various measurement tools for social capital have been developed such as the Social Capital Assessment Tools and its adapted version [42], the Social Capital Integrated Questionnaire (SCIQ) [43], Hurtado et al.’s six item tool [44], and the GSCS evaluated in the current study [32]. These tools emerged with the increase in the heterogeneous definition of social capital in many fields [45,46]. To the best of our knowledge, however, none of the scales have been used in the Chinese context, and the majority of studies were based on modified questions about the dimensions of social capital [47,48,49]. Thus, the present study recognizes the urgent need to establish the Chinese version of the standard scale for social capital.

Internal consistency and split-half reliability of the GSCS were analyzed, and results showed that all scales and subscales had acceptable internal consistency (>0.60) and split-half reliability except for the F3 (*feelings of trust and safety*) and F5 (*family and friend connections*) sub-scale. After a review of the said items, the problem in F3 may be caused by the differences between *feeling of trust* and *feeling of safety*. The original scale was built among communities, and the association between trust and safety has been identified in a previous study [50]. However, the sample of the current study consists of medical professionals, who may experience different feelings about safety because of workplace-specific problems such as violence [51,52]. In addition, the problems about F5 (*family and friend connections*) may be caused by the differences in the context between family and friends. The split-half reliability of the said sub-scale may be reduced because of the different connections between China and Western countries, where the family connection is more important in Chinese traditional culture [53].

The EFA results showed that the nine components obtained an acceptable cumulative rate, whereas the CFA results further supported the factor structure. However, item 13 indicated a higher factor loading in the *work connections* sub-scale. Item 13 (At work, do you take the initiative to do what needs to be done even if no one asks you to?) pertains to work, which may lead to its high factor loading in the *work connections* sub-scale. To address this issue, omission of the phrase “at work” is suggested.

The results of the present study support the notion that high levels of social capital are negatively associated with physical diseases and psychological distress. In fact, many previous studies have supported such an association in the Chinese and other contexts [54,55,56]. However, to the best of our knowledge, this association has not been identified among employees in China, especially medical professionals, as the majority of previous studies were conducted among communities and patients [57,58,59]. The results of the current study can add to the knowledge about associations between physical diseases, psychological distress, and social capital among Chinese medical professionals.

## 5. Conclusions

Data from a large Chinese sample of medical professionals supported the reliability and validity of the Chinese version of the GSCS, where high levels of social capital are negatively associated with physical diseases and psychological distress. Further studies that employ nationwide samples are required to replicate the current findings and further examine health-related outcomes of the Chinese version of the GSCS. By doing so, we wish to translate an applicable tool of a standard measure in the quantitative research on social capital to ensure the conclusive findings on social capital and its outcomes.

Certain limitations should be considered when interpreting the findings of the current study. First, the study was based on a cross-sectional design; thus, causal relations among the variables analyzed cannot be inferred. Second, certain indicators of reliability and validity were not calculated such as test–retest reliability and criterion validity due to limited data. Third, physical diseases and psychological distress were assessed via self-report. Thus, other accurate methods such as clinical evaluation may be helpful in obtaining factual conditions about the two variables.

Our findings identify social capital and other factors associated with health-related outcomes and should be helpful in the consideration of effective policies and interventions for physical and mental health of special occupational groups like medical professionals.

## Figures and Tables

**Table 1:** Social-demographic characteristics, chronic disease, and psychological distress of the sample.

Variable	*n* (Mean)	% (SD)
Total	3367	100.0
Gender		
Male	903	26.8
Female	2464	73.2
Age	35.12	8.43
Married Status		
Single	570	16.9
Married	2751	81.7
Others	46	1.4
Types of Medical Staff		
Doctor	1244	36.9
Nurse	1669	49.6
Medical Technician	454	13.5
Education		
Doctor	56	1.7
Master	556	46.5
Bachelor	2324	69.0
Others	431	12.8
Professional title		
Senior	109	3.2
Vice-senior	300	8.9
Intermediate	1139	33.8
Junior and others	1819	54.0
Manager		
Yes	649	19.3
No	2718	80.7
Chronic disease		
Yes	449	13.3
No	2918	86.7
Psychological distress	22.17	7.44

Note: SD refers to standard deviation.

**Table 2:** The internal consistency reliability and the split-half reliability for the GSCS-Chinese version (*n* = 3367).

Scale/Sub-Scale	Number of Items	Cronbach’s Alpha	Guttman Split-Half Coefficient
GSCS	34	0.920	0.752
F1: Participation in the Local Community	7	0.908	0.869
F2: Social Agency or Proactivity in a Social Context	7	0.817	0.753
F3: Feelings of Trust and Safety	5	0.747	0.629
F4: Neighborhood Connections	5	0.813	0.763
F5: Family and Friends Connections	3	0.698	0.604
F6: Tolerance of Diversity	2	0.872	0.872
F7: Value of Life	2	0.692	0.692
F8: Work Connections	3	0.807	0.726

**Table 3:** Exploratory factor analysis for the factors structure of the GSCS-Chinese version.

Items	F1	F2	F3	F4	F5	F6	F7	F8	F9
Item 1	**0.55**	0.25	0.00	0.12	0.10	0.04	−0.09	0.02	0.20
Item 2	**0.79**	0.07	0.05	0.01	0.09	0.12	0.01	0.03	−0.03
Item 3	**0.82**	0.01	0.07	−0.05	0.11	0.05	0.03	0.08	−0.09
Item 4	**0.83**	−0.01	0.05	−0.01	0.10	0.02	0.02	0.09	−0.10
Item 5	**0.84**	0.09	0.03	0.04	0.11	0.02	0.02	0.04	0.03
Item 6	**0.83**	0.08	0.03	0.05	0.12	0.00	0.02	0.04	0.05
Item 7	**0.83**	0.07	0.03	0.01	0.15	0.00	0.00	0.08	−0.04
Item 8	0.14	**0.69**	−0.11	0.16	0.17	0.08	0.02	−0.14	0.15
Item 9	0.13	**0.68**	0.02	0.13	0.12	0.17	0.03	0.01	0.13
Item 10	0.13	**0.65**	0.13	0.18	0.01	0.13	0.14	0.21	0.03
Item 11	0.10	**0.62**	0.24	−0.04	0.12	0.04	0.17	0.27	0.07
Item 12	0.09	**0.62**	0.27	−0.09	0.09	0.08	0.11	0.26	0.09
Item 13	−0.15	**0.50**	0.27	0.09	0.01	0.02	0.17	−0.15	**0.51**
Item 14	−0.05	**0.50**	0.27	0.06	0.11	0.13	0.13	−0.14	0.45
Item 15	0.06	0.19	**0.69**	0.34	0.05	0.11	0.11	−0.09	0.18
Item 16	0.04	0.25	**0.67**	0.26	0.11	0.11	0.07	0.09	0.28
Item 17	0.26	0.12	**0.56**	−0.03	0.30	0.03	−0.04	0.29	0.00
Item 18	0.08	0.11	0.24	**0.77**	0.11	0.12	0.11	0.14	0.14
Item 19	0.00	0.16	0.12	**0.77**	0.21	0.05	0.12	0.20	0.18
Item 20	0.00	0.20	0.14	0.37	**0.45**	0.21	0.09	0.10	0.34
Item 21	0.19	0.05	0.19	0.13	**0.70**	0.07	0.01	0.21	0.03
Item 22	0.28	0.06	0.07	−0.02	**0.77**	0.12	0.05	0.15	0.00
Item 23	0.08	0.15	0.05	0.17	**0.67**	0.18	0.15	−0.04	0.22
Item 24	0.26	0.15	0.01	0.07	**0.73**	0.10	0.04	0.06	0.07
Item 25	0.05	0.17	0.07	0.09	0.21	**0.74**	0.07	0.08	0.16
Item 26	0.03	0.11	0.06	0.09	0.06	**0.76**	0.09	0.08	0.21
Item 27	0.14	0.17	0.06	0.02	0.22	**0.63**	0.29	0.16	−0.01
Item 28	−0.02	0.22	0.06	0.14	0.10	0.20	**0.81**	0.10	0.27
Item 29	0.00	0.19	0.08	0.14	0.11	0.21	**0.81**	0.13	0.25
Item 30	0.17	0.16	0.08	0.23	0.19	0.24	0.24	**0.59**	0.22
Item 31	0.17	0.10	0.06	0.20	0.19	0.14	0.05	**0.68**	0.18
Item 32	0.10	0.14	0.04	0.21	0.20	0.10	0.16	0.45	**0.56**
Item 33	0.01	0.12	0.12	0.10	0.12	0.20	0.15	0.20	**0.77**
Item 34	−0.08	0.17	0.11	0.15	0.06	0.14	0.20	0.15	**0.79**

Note: The bold numbers mean the highest factor loading in all the factors.

**Table 4:** Logistic regression for the effect of general social capital on physical health.

Variables	OR	95% CI		*p*
		Lower	Upper	
Male	1.24	0.95	1.63	0.114
Age	1.05	1.04	1.07	<0.001
Married Status (Ref. = Others)				
Single	2.19	0.72	6.67	0.168
Married	2.35	0.82	6.73	0.110
Types of Medical Staff (Ref. = Medical technician)				
Doctor	0.97	0.69	1.36	0.859
Nursing	1.12	0.79	1.58	0.531
Education (Ref. = Others)				
Doctor	1.30	0.53	3.15	0.567
Master	1.29	0.79	2.09	0.311
Bachelor	1.67	1.13	2.48	0.010
Professional Title (Ref. = Junior and Others)				
Senior	2.41	1.29	4.50	0.006
Vice-senior	1.99	1.25	3.16	0.003
Intermediate	1.67	1.24	2.24	0.001
Manager	1.08	0.82	1.44	0.573
GSCS	0.98	0.98	0.99	<0.001
Constant	0.02			<0.001
R^2^ = 0.123				

Note: GSCS = general social capital scale; OR = odds ratio; CI = confidence interval.

**Table 5:** OLS regression for the effect of general social capital on psychological distress.

Variables	β	95% CI		*p*
		Lower	Upper	
Male	0.04	0.08	1.35	0.027
Age	0.06	0.01	0.10	0.022
Married Status (Ref. = Others)				
Single	−0.05	−1.66	−0.13	0.022
Married	0.03	−1.66	2.71	0.638
Types of Medical Staff (Ref. = Medical technician)				
Doctor	0.04	−1.33	2.84	0.477
Nursing	−0.03	−4.08	0.04	0.055
Education (Ref. = Others)				
Doctor	0.05	0.08	2.07	0.035
Master	0.05	0.09	1.58	0.029
Bachelor	0.11	0.92	2.52	<0.001
Professional Title (Ref. = Junior and Others)				
Senior	0.10	0.73	2.30	<0.001
Vice-senior	0.01	−1.48	2.07	0.743
Intermediate	−0.01	−1.58	0.88	0.580
Manager	0.04	−0.03	1.34	0.060
GSCS	−0.28	−0.15	−0.12	<0.001
Constant	29.55	26.50	32.61	<0.001
R^2^ = 0.094				

Note: GSCS = general social capital scale; CI = confidence interval.

## Data Availability

The datasets used and/or analyzed during the current study are available from the corresponding author on reasonable request.
